# Human stem cell aging: do mitochondrial DNA mutations have a causal role?

**DOI:** 10.1111/acel.12199

**Published:** 2014-01-28

**Authors:** Holly L Baines, Douglass M Turnbull, Laura C Greaves

**Affiliations:** 1 Centre for Brain Ageing and Vitality, Institute for Ageing and Health, The Medical SchoolNewcastle upon Tyne, NE2 4HH, UK; 2 Wellcome Trust centre for Mitochondrial Research, Institute for Ageing and Health, Newcastle UniversityNewcastle upon Tyne, NE2 4HH, UK

**Keywords:** aging, human, mitochondria, mutator mouse, stem cells

## Abstract

A decline in the replicative and regenerative capacity of adult stem cell populations is a major contributor to the aging process. Mitochondrial DNA (mtDNA) mutations clonally expand with age in human stem cell compartments including the colon, small intestine, and stomach, and result in respiratory chain deficiency. Studies in a mouse model with high levels of mtDNA mutations due to a defect in the proofreading domain of the mtDNA polymerase γ (mtDNA mutator mice) have established causal relationships between the accumulation of mtDNA point mutations, stem cell dysfunction, and premature aging. These mtDNA mutator mice have also highlighted that the consequences of mtDNA mutations upon stem cells vary depending on the tissue. In this review, we present evidence that these studies in mice are relevant to normal human stem cell aging and we explore different hypotheses to explain the tissue-specific consequences of mtDNA mutations. In addition, we emphasize the need for a comprehensive analysis of mtDNA mutations and their effects on cellular function in different aging human stem cell populations.

## Introduction

### Stem cell aging

Aging is a stochastic process characterized by a decline in the homeostatic and regenerative processes within tissues (Kirkwood, 2005). Common features of aging such as decreased wound healing in the skin, decreased immunity, hair greying, and hair loss all result from reduced tissue homeostasis and regeneration with age, due to diminished somatic stem cell function (Sharpless & DePinho, 2007).

Under normal conditions, stem cells divide asymmetrically to give another stem cell for self-renewal and one daughter cell that differentiates into an effector/progenitor cell (Liu & Rando, 2011) to maintain normal tissue function. As we age, stem cells incur damage due to features of both chronological and replicative aging, especially stem cells in rapidly replicating tissues such as the gut, skin, and blood (Liu & Rando, 2011). In some replicating tissues, this causes depletion in the stem cell pool, either due to replicative senescence or a decline in the self-renewal capacity, commonly seen in neural stem cells (Maslov *et al*., 2004) and melanocyte stem cells (Nishimura *et al*., 2005). Alternatively, stem cells may undergo malignant transformation, increasing the risk of cancer with age (Liu & Rando, 2011). However, in the majority of cases, stem cells endure alterations in their normal fate and function of their progeny due to disturbances in differentiation, as seen in hematopoietic stem cells (HSCs) (Rossi *et al*., 2005). Understanding the molecular mechanisms responsible for such age-related changes in normal fate and function of adult stem cell populations is important if we ever aim to deter tissue degeneration, prolong regeneration, and improve healthy aging.

Damage to the mitochondrial DNA (mtDNA) resulting in respiratory chain dysfunction has been proposed to be a significant contributor to the aging phenotype (Linnane *et al*., 1989). Numerous studies present evidence for a relationship between mtDNA defects and human stem cell aging (Taylor *et al*., 2003; McDonald *et al*., 2008; Fellous *et al*., 2009); however, this data may just be circumstantial and mtDNA defects could merely be a biomarker of the aging process. Here, we discuss the significance of mtDNA point mutations and respiratory chain deficiency in aging human mitotic tissues and evaluate studies performed in the mtDNA mutator mouse to determine a causal role for mitochondria in normal stem cell aging.

## Mitochondria

Mitochondria are dynamic, intracellular organelles that primarily function to generate ATP by the process of oxidative phosphorylation. The mitochondrial genome is the only extra nuclear source of DNA within a cell and is a covalently closed, 16 596 base pair molecule, encoding 37 genes including 13 essential subunits of the respiratory chain, 2 rRNAs (12s and 16s), and 22 tRNAs (Anderson *et al*., 1981).

### Mitochondria and aging

The mitochondrial theory of aging is based upon the principle that somatic mutations in mtDNA accumulate throughout life and result in reduced mitochondrial and respiratory chain function (Miquel *et al*., 1980). Tissue dysfunction subsequently arises from defects in energy metabolism, apoptosis, and senescence, eventually leading to age-related frailty and disease (Taylor & Turnbull, 2005). The mitochondrial free radical theory of aging suggests that this activates a vicious cycle, whereby dysfunction of the oxidative phosphorylation system causes enhanced ROS production and further damage to mtDNA, leading to the progressive accumulation of somatic mtDNA mutations with age and further oxidative damage, eventually resulting in cell death (Harman, 1972). However, there is much controversy surrounding the mitochondrial free radical theory of aging, as long-lived species do not always demonstrate lower levels of ROS (Chen *et al*., 2007) and long-lived mammals, such as the naked mole rat, demonstrate fairly high levels of ROS and high levels of oxidative damage to lipids, DNA, and proteins (Andziak *et al*., 2006). Moreover, there is no evidence to suggest that the incidence of oxidative damage to mtDNA is greater than nuclear DNA (Lim *et al*., 2005).

Alternatively, it has been proposed that mtDNA mutations are principally induced by replication errors mediated by mtDNA polymerase Y (Zheng *et al*., 2006). Data from computational models (Elson *et al*., 2001) and mitochondrial mutation assays (Coller *et al*., 2005) suggest that such replication errors arise during early development, accumulating throughout life by the mechanism of clonal expansion and random genetic drift (Elson *et al*., 2001). Given the multicopy nature of mtDNA within cells, most point mutations are highly recessive and it is only when a point mutation clonally expands and reaches a critical threshold level that a respiratory chain defect is observed. This threshold differs according to the type of mtDNA mutation but is generally ~60% for deleted mtDNA (Sciacco *et al*., 1994) and ~85% for point mutations (Boulet *et al*., 1992). This defect can be readily identified by the absence of histochemical staining for cytochrome c oxidase (COX) (Old & Johnson, 1989).

## Somatic MtDNA mutations in aging human mitotic tissues

MtDNA mutations were initially detected in aging human postmitotic tissues, with a mosaic pattern of COX deficiency seen in the heart (Muller-Hocker, 1989), muscle (Muller-Hocker, 1990), and brain (Cottrell *et al*., 2001). More recently, respiratory chain defects have been shown to accumulate to significant levels in aging human mitotic tissues, including the colon (Taylor *et al*., 2003), liver, pancreas (Fellous *et al*., 2009), stomach (McDonald *et al*., 2008), and small intestine (Gutierrez-Gonzalez *et al*., 2009). Analysis of single COX-deficient cells in these studies revealed that the cellular defect was directly caused by clonally expanded somatic mtDNA point mutations. In the human colon, COX-deficient crypts are rarely detected below the age of 30 (Taylor *et al*., 2003) and the majority of mtDNA point mutations identified are base transitions, which are likely to be due to errors during mtDNA replication (Greaves *et al*., 2012). This further supports the hypothesis that somatic mtDNA mutations principally arise during early life and accumulate throughout adulthood by clonal expansion (Elson *et al*., 2001; Coller *et al*., 2005). Furthermore, in the aging, human colon somatic mtDNA mutations occur at random and are commonly nonsynonymous or frameshift mutations that are significantly more pathogenic than germline variants (Greaves *et al*., 2012). This indicates an absence of selective constraints, replication, and/or selective clonal expansion of somatic mtDNA mutations in mitotic tissues. In mitotic tissues, the only long-lived cells are the stem cells, and so, the mtDNA mutations must be occurring and being fixed in these cells, strongly indicating a role for mtDNA mutations in stem cell aging.

In the aging, human colon mtDNA mutations and respiratory chain deficiency have been associated with an altered cellular phenotype characterized by a decrease in crypt cell number and cell proliferation (Nooteboom *et al*., 2010). However, this is the only study to have explored the functional consequences of mitochondrial dysfunction in a replicating human tissue as studies in human subjects are limited due to the availability of human tissue samples and a lack of robust stem cell markers. Therefore, it remains largely unknown how mtDNA defects contribute to human stem cell aging.

## Use of animal models

To explore the potential role of mtDNA point mutations in aging, it is therefore necessary to use animal models. The development of the mtDNA mutator mouse provided definitive evidence for a causal relationship between mtDNA mutations and aging. These mice have a homozygous mutation (D257A) in the proofreading domain of mtDNA polymerase γ, significantly reducing its proofreading activity and resulting in the accelerated accumulation of mtDNA mutations and severe respiratory chain deficiency (Trifunovic *et al*., 2004; Kujoth *et al*., 2005). MtDNA mutator mice display a reduced lifespan and a premature aging phenotype, which closely resembles normal human aging and is characterized by kyphosis, reduced subcutaneous fat, hair loss, weight loss, anemia, osteoporosis, reduced fertility, and enlargement of the heart (Trifunovic *et al*., 2004; Kujoth *et al*., 2005).

### Premature aging is driven by stem cell dysfunction in mtDNA mutator mice

Somatic mtDNA mutations in the mtDNA mutator mice arise from replication errors during development (Ameur *et al*., 2011), and a number of studies have shown that this causes early-onset stem cell dysfunction, which drives the tissue-specific aging phenotypes in these mice.

Abnormalities in stem cell populations in the mtDNA mutator mice vary significantly depending on the tissue; with some displaying a direct effect upon the stem cell pool and others demonstrating effects on downstream differentiation events and early precursors. In the hematopoietic system of mtDNA mutator mice, mtDNA mutations induce blocks in early differentiation events and cause abnormalities in downstream hematopoietic progenitor cells, but have no direct effect on the hematopoietic stem cell (HSC) pool (Norddahl *et al*., 2011). The impaired differentiation and resulting abnormal myeloid lineages are directly associated with anemia and lymphopenia in the mtDNA mutator mice, which are the principal driving factors of premature aging in these animals (Ahlqvist *et al*., 2012). By comparison, abnormalities in neural stem cells show a direct effect upon stem cell function, distinguished by a decline in the number of quiescent stem cells and a decrease in their self-renewal capacity in response to mtDNA mutation accumulation (Ahlqvist *et al*., 2012). Studies of the epithelial crypt cells in the small intestine of mtDNA mutator mice demonstrated increased levels of apoptosis, a decrease in the number of proliferating cells, and impaired development of stem cell-derived organoids *in vitro* (Fox *et al*., 2012). These abnormalities corresponded with the loss of respiratory chain activity and contributed to reduced absorption of dietary lipids in the gut (Fox *et al*., 2012).

Taken together, these studies indicate that aging is driven by stem cell dysfunction in response to the accumulation of mtDNA point mutations. However, it still remains unknown exactly how mtDNA point mutations cause stem cell dysfunction and moreover why we see tissue-specific alterations in either the stem cells directly or stem cell fate and downstream differentiation events? We know that there are different cell fates for damaged stem cells; however, we do not know what determines whether a damaged stem cell will undergo cell death, senescence, or malignant transformation in response to mtDNA mutations.

### Proposed mechanisms for the effect of somatic mtDNA mutations on stem cell function

**Figure 1:**
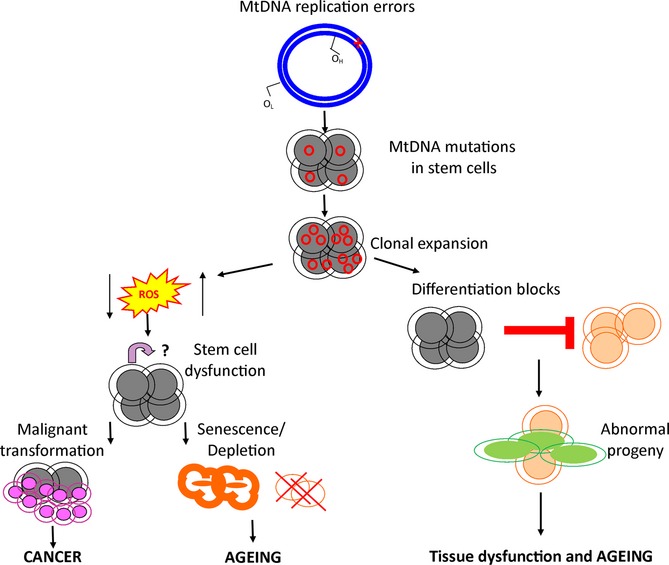
Schematic diagram hypothesizing the possible mechanisms by which mtDNA point mutations may affect stem cell function and drive aging phenotypes. MtDNA mutations arise in stem cells during early development due to errors during mtDNA replication and accumulate throughout life by clonal expansion. Upon reaching a critical threshold level, mtDNA mutations may cause slight alterations in ROS signaling, affecting the quiescent state of stem cells and their capacity for regeneration and reconstitution. Depending on the active/quiescent state of stem cells, this may either lead to aberrant proliferation and malignant transformation or may cause depletion in the stem cell pool and a subsequent decline in tissue function. Alternatively, mtDNA mutations may have no direct effect upon the stem cells but may instead act at the point of early differentiation, causing differentiation blocks and the production of abnormal progeny cells, contributing to a loss of normal tissue homeostasis and age-related dysfunction.

One plausible explanation is the individual stem cell niche and microenvironment. The maintenance of somatic stem cells relies upon a balance between self-renewal and differentiation, which is regulated partly by signaling and physiological ROS molecules (Hamanaka and Chandel, 2010). Alterations in ROS signaling have major effects upon the quiescent/active state of stem cell populations causing shifts in proliferation or differentiation, particularly in HSCs (Shao *et al*., 2011). In mtDNA mutator mice, the abnormal phenotypes observed in neural stem cells and hematopoietic progenitor cells were rescued by supplementation with the antioxidant N-acetyl-L-cysteine (Ahlqvist *et al*., 2012). This implies that mtDNA mutations can cause slight alterations in redox status to which stem cells are highly sensitive to, thus affecting their capacity for regeneration and reconstitution (Ahlqvist *et al*., 2012). It is worth acknowledging that N-acetyl-L-cysteine is a widely used pharmaceutical and is involved in numerous physiological processes other than just ROS scavenging, some of which include modulating cell proliferation, regulating the immune response and metabolism of prostaglandins and leukotrienes (Samuni *et al*., 2013). Thus, N-acetyl-L-cysteine treatment could have reversed the abnormal stem cell phenotypes in the mtDNA mutator mice by affecting other physiological mechanisms other than just redox status. Nevertheless, it is highly plausible that mtDNA mutations may cause mild alterations in ROS signaling and that it is the specific stem cell niche and whether or not the stem cells are in an active or quiescent state that determines how a stem cell will respond to the change in redox status and the subsequent change in cell fate and tissue-specific dysfunction (Fig. 1). For example in the gut, a highly proliferative tissue, the stem cells are fairly active and so alterations in ROS signaling are likely to affect proliferation events, as seen in the mtDNA mutator mouse (Fox *et al*., 2012). This is in contrast to the hematopoietic system where HSCs are maintained in a fairly dormant/quiescent state (Shao *et al*., 2011) and changes in ROS signaling may only take effect at the point of stem cell activation and differentiation, as seen in the mtDNA mutator mice (Norddahl *et al*., 2011; Ahlqvist *et al*., 2012).

Alternatively, mtDNA mutations may not directly affect stem cell function but may only act at the point of early stem cell differentiation, leading to the production of abnormal progeny cells and a loss of somatic tissue maintenance (Fig. 1). In mtDNA mutator mice, mtDNA point mutations were found to affect hematopoietic progenitor cells during differentiation, and HSCs remained unaffected despite presenting with the same level of mtDNA mutations (Norddahl *et al*., 2011; Ahlqvist *et al*., 2012). Evidence suggests that somatic stem cells may be quite resistant to mitochondrial dysfunction as they are maintained in a fairly dormant/quiescent state, contain few mitochondria, and rely more on glycolysis for energy production than oxidative metabolism (Simsek *et al*., 2010; Shao *et al*., 2011). It is only during differentiation that mitochondrial content and ATP levels increase, suggesting that oxidative phosphorylation plays only a minor role in quiescent stem cells and self-renewal, but downstream progenitors require intact mitochondrial function (Inoue *et al*., 2010). Thus, it may be the tissue-specific differentiation processes of stem cells and the sensitivity of progeny cells that govern the different aging phenotypes caused by mtDNA point mutations.

## Concluding remarks

In this review, we have presented evidence that somatic mtDNA mutations and respiratory chain deficiency accumulate to significant levels in aging human replicative tissues, and evidence from the mtDNA mutator mice demonstrates that mtDNA mutations directly cause stem cell dysfunction and induce age-related phenotypes. It has been shown that somatic mutagenesis is influenced by pre-existing germline mtDNA mutations in mice that can accelerate the clonal expansion of somatic mtDNA mutations and have life-long consequences, causing certain features of premature aging (Ross *et al*., 2013). Low-level heteroplasmic mtDNA mutations are also commonly inherited through the female germline in humans (Li *et al*., 2010; Payne *et al*., 2013) and are thus likely to aggravate somatic mutagenesis and the aging process in subsequent generations.

Studies in the mtDNA mutator mice have also highlighted the importance of interactions between the nuclear and mitochondrial genome, when considering the role of somatic mtDNA mutations in aging. In the two strains of mtDNA mutator mice, the different nuclear backgrounds appear to play a significant role in the severity of the premature aging phenotypes, the levels of COX deficiency detected in tissues (H.L. Baines & L.C. Greaves, unpublished data from our lab), and lifespan, with the European strain (C57Bl/6N background) (Trifunovic *et al*., 2004) displaying a more severe phenotype than the American strain (C57Bl/6J background) (Kujoth *et al*., 2005). Thus, it is likely that variation between human nuclear genomes will influence the effects and extent to which somatic mtDNA mutations and mitochondrial dysfunction play a role in tissue dysfunction and aging.

While the incidence of mitochondrial dysfunction is higher in the mtDNA mutator mice than in normal human aging, the development and types of mtDNA mutation, the clonal expansion, and the respiratory chain deficiency are similar. Thus, somatic mtDNA mutations are likely to contribute to stem cell dysfunction and age-related phenotypes in a similar manner in normal human aging, but to a lesser effect. Nevertheless, in mtDNA mutator mice, aging is driven solely by mtDNA mutations, whereas normal human aging is a complex, multifactorial process driven by multiple molecular mechanisms including not only mtDNA defects but also ROS, senescence, and damaged proteins.

To truly understand the role of mtDNA point mutations in human stem cell aging, future research should focus on establishing a comprehensive overview of clonally expanded mtDNA point mutations and respiratory chain deficiency in all aging human replicative tissues, in particular concentrating on any associated changes in markers of stem cell function such as proliferation, differentiation, and self-renewal. Furthermore, studies should be performed in replicative tissues of mitochondrial disease patients with different mtDNA point mutations as this would have profound implications for our understanding of mutation and tissue-specific effects in stem cell compartments. Only then will we be able to determine the consequences and contribution of mtDNA defects in stem cell aging and how this may influence the human aging phenotype as a whole.
